# Prognostic value of microRNA-125a expression status in molecular groups of pediatric medulloblastoma

**DOI:** 10.1007/s00381-023-05899-z

**Published:** 2023-03-09

**Authors:** Soheir M. Hamam, Eman Abdelzaher, Shady H. Fadel, Rasha A. Nassra, Hend A. Sharafeldin

**Affiliations:** 1 Pathology department, Faculty of Medicine, Alexandria University, Alexandria, Egypt; 2 Pediatric Oncology and Nuclear Medicine, Faculty of Medicine, Alexandria University, Alexandria, Egypt; 3 Medical Biochemistry, Faculty of Medicine, Alexandria University, Alexandria, Egypt

**Keywords:** WNT, SHH, Non-WNT/non-SHH, MicroRNA-125a

## Abstract

**Purpose:**

Medulloblastoma (MB) is the most common malignant pediatric brain tumor. Current treatment allows decent survival rates but often with life-long morbidity. Molecular classification provides a base for novel therapeutic approaches. However, these groups are heterogeneous. MicroRNA-125a has a tumor suppressor function. It is downregulated in several tumors. The expression of microRNA-125a in MB patients remains unclear. Therefore, this study was designed to evaluate the expression of microRNA-125a in molecular groups of pediatric MB patients in Egyptian population and its clinical significance.

**Methods:**

Formalin-fixed, paraffin-embedded tissue blocks from 50 pediatric MB patients were retrospectively collected. Immunohistochemistry for β-catenin, GAB1, YAP1, and p53 was done for molecular classification. MicroRNA-125a expression analysis was done using qRT-PCR. Follow-up data were obtained from patients’ records.

**Results:**

MicroRNA-125a expression was significantly lower in MB patients showing large cell/anaplastic (LC/A) histology and in the non-WNT/non-SHH group. Lower levels of microRNA-125a showed a tendency toward poor survival rates; however, difference was not significant. Infants and larger preoperative tumor size were significantly associated with lower survival rates. On a multivariate analysis, preoperative tumor size was an independent prognostic factor.

**Conclusion:**

MicroRNA-125a expression was significantly lower in categories of pediatric MB patients with worse prognosis namely LC/A histology and the non-WNT/non-SHH group suggesting a pathogenetic role. MicroRNA-125a expression could represent a promising prognostic factor and a potential therapeutic target in the non-WNT/non-SHH group which represents the most common and the most heterogeneous group of pediatric MBs coupled with the highest rates of disseminated disease. Preoperative tumor size represents an independent prognostic factor.

## Introduction

Medulloblastoma (MB) is the most common malignant pediatric brain tumor [1]. Children aged 4 to 9 years old show the highest incidence at 44% [2].

Studies of MB demonstrated that mutations in cell signaling pathways are associated with specific gene expression signatures, which could be detected using relatively simple and widely available techniques [3]. This has led to the introduction of the molecular subtyping in the 2016 World Health Organization (WHO) classification which is still endorsed by the 2021 WHO classification [4, 5].

According to the 2021 WHO classification of central nervous system (CNS) tumors, MBs are classified based on histopathological features and molecular characteristics. According to molecular characteristics, MB is classified into four groups: WNT (wingless-related integration site)-activated MBs, SHH (sonic hedgehog)-activated MBs which are separated into TP53-mutant and TP53-wild-types, and non-WNT/non-SHH MBs, comprising group 3 and group 4 which are listed as provisional variants [5].

The current treatment of MB allows 5-year survival rates ranging between 50 and 90%. This wide range is multifactorial depending on age at diagnosis, the presence of metastasis at diagnosis, histological pattern, and molecular group. Moreover, current therapeutic modalities come at the expense of life-long morbidity [6]. Therefore, the recent WHO classification has provided a base for novel therapeutic approaches that target cell signaling pathways that are activated aberrantly in MB [7]. However, these groups are heterogeneous, and they need more research to improve the molecular understanding and management of MB [8].

MicroRNAs are small noncoding RNAs that regulate gene expression through sequence-specific binding to 3′-untranslated regions of mRNAs, resulting in translational inhibition or mRNA degradation [9]. Some microRNAs show altered expression in many malignancies suggesting a critical role played by them in tumorigenesis [10].

The published literature largely lacks detailed studies of microRNA expression in MBs. A single study, conducted before the release of the 2016 WHO classification, focused on microRNA expression profiling in MB, where specific microRNA signatures distinguished tumors from normal cerebellar tissues [11].

MicroRNA-125a is located at 19q13. It is expressed in differentiated cells and acts as an antiproliferative factor. MicroRNA-125a expression is downregulated in several tumors [12]. MicroRNA-125a gene has two mature microRNAs in its precursor structure: hsa-mir-125a-5p and hsa-mir-125a-3p. MiR-125a-5p has a prognostic role in several tumors, and its mimic alone or in combination with other therapeutic agents could be a novel therapeutic approach against these tumors [13].

To date, the expression profile of microRNA-125a in MB patients remains unclear. Therefore, this study was designed to evaluate the expression of microRNA-125a in different molecular groups of pediatric MB patients in Egyptian population and its clinical significance.

## Methods

The current retrospective study included tumor samples from 50 archived formalin-fixed, paraffin-embedded (FFPE) tissue blocks obtained from MB patients up to the age of 18 years. Samples were obtained from the Pathology Department, Faculty of Medicine, Alexandria University, Egypt, during the period from 2009 to 2018 upon the approval of the ethics committee at the Faculty of Medicine, Alexandria University. None of the patients previously received chemotherapy or radiotherapy.

### Patient’s data

Clinical and follow-up data were obtained by reviewing patients’ records at the Clinical Oncology Department, Borg El-Arab Children’s Cancer Hospital, Alexandria University, Egypt. The date of assessment was January 2022.

Patients were classified into standard- and high-risk groups based on age at diagnosis, presence/absence of leptomeningeal dissemination (based on magnetic resonance imaging (MRI) and cerebrospinal fluid (CSF) analysis), extent of residual tumor after resection, and presence/absence of large cell/anaplastic (LC/A) histology. Standard-risk patients are children aged > 3 years without leptomeningeal dissemination, postoperative residual tumor < 1.5cm^2^, and non-LC/A histology [6].

Infants received HIT-SKK protocol with intensive chemotherapy followed by craniospinal radiotherapy at the age of 3 years [14].

Standard-risk patients were treated with whole CNS radiotherapy 23.4 Gy and boost up to 54 Gy with weekly vincristine. High-risk patients were treated with whole CNS radiotherapy 36 Gy and boost up to 54 Gy. Both standard- and high-risk patients received adjuvant chemotherapy (8 cycles of cisplatin, Endoxan, and vincristine) [15–17].

### Histopathologic examination

Diagnosis was made conforming to the WHO criteria of 2021 [5].

### Immunohistochemistry (IHC)

It was performed on 4-μm FFPE tissue sections using Dako PT Link unit and Dako Autostainer (Link 48, Agilent Technologies Inc., CA, USA).

For molecular classification, IHC for β-catenin antibody, a mouse monoclonal antibody (clone 15B8, 14–2567-82, Thermo Fischer Scientific, CA, USA), GAB1 antibody, a rabbit polyclonal antibody (GTX111253, GeneTex, CA, USA), YAP1 antibody, a mouse monoclonal antibody (G-6, sc-376830, Santa Cruz Biotechnology Inc., CA, USA), and p53 (DO-7, Leica Biosystems Inc., IL, USA) was done [18].

WNT group was identified by positive nuclear staining of β-catenin (≥ 5%) [19] and YAP1 nuclear positivity, while the SHH group was identified by positive cytoplasmic immunostaining of GAB1 (≥ 25%) [18] and YAP1 nuclear positivity. Non-WNT/non-SHH tumors were identified by negative nuclear β-catenin, GAB1, and YAP1 staining [20]. P53 immunostaining was performed in the SHH group where widespread strong nuclear positivity was considered as TP53-mutant, while scattered weak nuclear positivity was considered as TP53-wild-type [5].

For cases of LC/A histology, synaptophysin and integrase interactor 1 (INI 1) IHC were performed to confirm the diagnosis.

### Histochemical staining for reticulin

It was performed in cases of histologically detected nodules to differentiate between desmoplastic nodular MB (DNMB) and biphasic pattern of classic MB.

### Quantitative reverse transcription-polymerase chain reaction (qRT-PCR)

1. Expression profile analysis of microRNA and quantitative real-time PCR
  1.1. RNA extraction from FFPE tissue samples
    Samples were deparaffinized using deparaffinization solution (cat. no. 19093) and protease digested; then, total RNA isolation from samples was performed using QIAGEN® RNeasy FFPE Kit (ID: 73,504) according to the manufacturer’s instructions.
    The RNA concentration and purity were measured using NanoDrop 2000/2000c spectrophotometer (Thermo Scientific, USA).
  1.2. Real-time qRT-PCR
    Complementary DNA (cDNA) was synthesized using TaqMan ®MicroRNA Reverse Transcription Kit with miRNA specific primers (Applied Biosystems, USA).
    RT reactions for target (microRNA-125a) and spiked-in control (RNU6B) were performed. In brief, 10 ng of RNA, specific stem-loop primers for each miRNA, 10 × buffer, dNTPs, reverse transcriptase, and RNase inhibitor were loaded in a thermal cycler (Primus 25 advanced, PEQLAB, UK) following the manufacturer’s protocol.
    Then real-time PCR was carried out using the miRNA-specific TaqMan probes and TaqMan universal master mix in Applied Biosystems StepOne™ Real-Time PCR System. The expression levels of the miRNA-125a in each sample were normalized to those of RNU6B. The arithmetic formula (2^−ΔCT^) was used to calculate the median and range for each group as individual data points, and the difference in expression between patient groups was assessed [21].

### Statistical analysis

Data were analyzed using the Statistical Package for Social Sciences (SPSS ver. 25 Chicago, IL, USA) [22]. Descriptive statistics were summarized for categorical variables as frequencies and percentages and for continuous variables as mean (*M*) and median (*Mdn*). Various tests were used including Mann–Whitney (*U*), Kruskal–Wallis (*H*), Pearson’s correlation (*r*), and Spearman’s rho correlation coefficient (*ρ*).

Overall survival (OS) was defined as the time from diagnosis until death or last visit. Event-free survival (EFS) was defined as the time from diagnosis until disease recurrence/progression or death. Log rank test was used to compare the patients’ outcome between different groups. Univariate Cox regression was used to evaluate the effect of continuous covariates on patients’ outcome.

For the multivariate analysis, Cox survival regression analysis was used. For each variable, hazard ratio (HR) was calculated, and its 95% confidence interval (CI) was determined. In all statistical tests, level of significance of 0.05 was used, which the results were considered to be statistically significant.

## Results

### Clinicopathological characteristics

The current study included 50 MB patients: 32 (64%) were males, and 18 (36%) were females. Age of the patients ranged between 2 and 18 years (*M* = 9.7, ± 5.1 years); 5 (10%) were ≤ 3 years, and 45 (90%) were > 3 years; 22 patients (44%) were standard risk, and 28 patients (56%) were high risk.

**Fig. 1:**
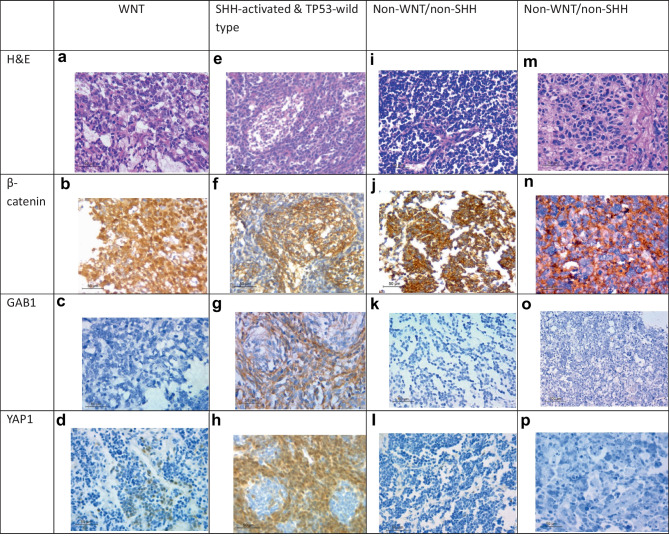
Histopathological and immunohistochemical results of MB cases. WNT MB **a** classic histology (H&E × 400), **b** β-catenin positive nuclear staining (anti β-catenin X400), **c** GAB1 negative staining (anti GAB1 X400) **d** YAP1 positive nuclear staining (anti YAP1 X400); SHH-activated & TP53-wildtype MB **e** desmoplastic nodular histology (H&E X400) **f** β-catenin cytoplasmic staining (anti β-catenin X400) **g** GAB1 positive cytoplasmic staining (anti GAB1 X400) **h** YAP1 internodular positive nuclear staining and scattered positive intranodular cells (anti YAP1 X400); non-WNT/non-SHH MB **i** classic histology (H&E X400) **j** β-catenin cytoplasmic staining (anti β-catenin X400) **k** GAB1 negative staining (anti GAB1 X400) **l** YAP1 negative staining (anti YAP1 X400); non-WNT/non-SHH MB **m** LC/A histology (H&E X400) **n** β-catenin cytoplasmic staining (anti β-catenin X400) **o** GAB1 negative staining (anti GAB1 X200) **p** YAP1 negative staining (anti YAP1 X400)

**Fig. 2:**
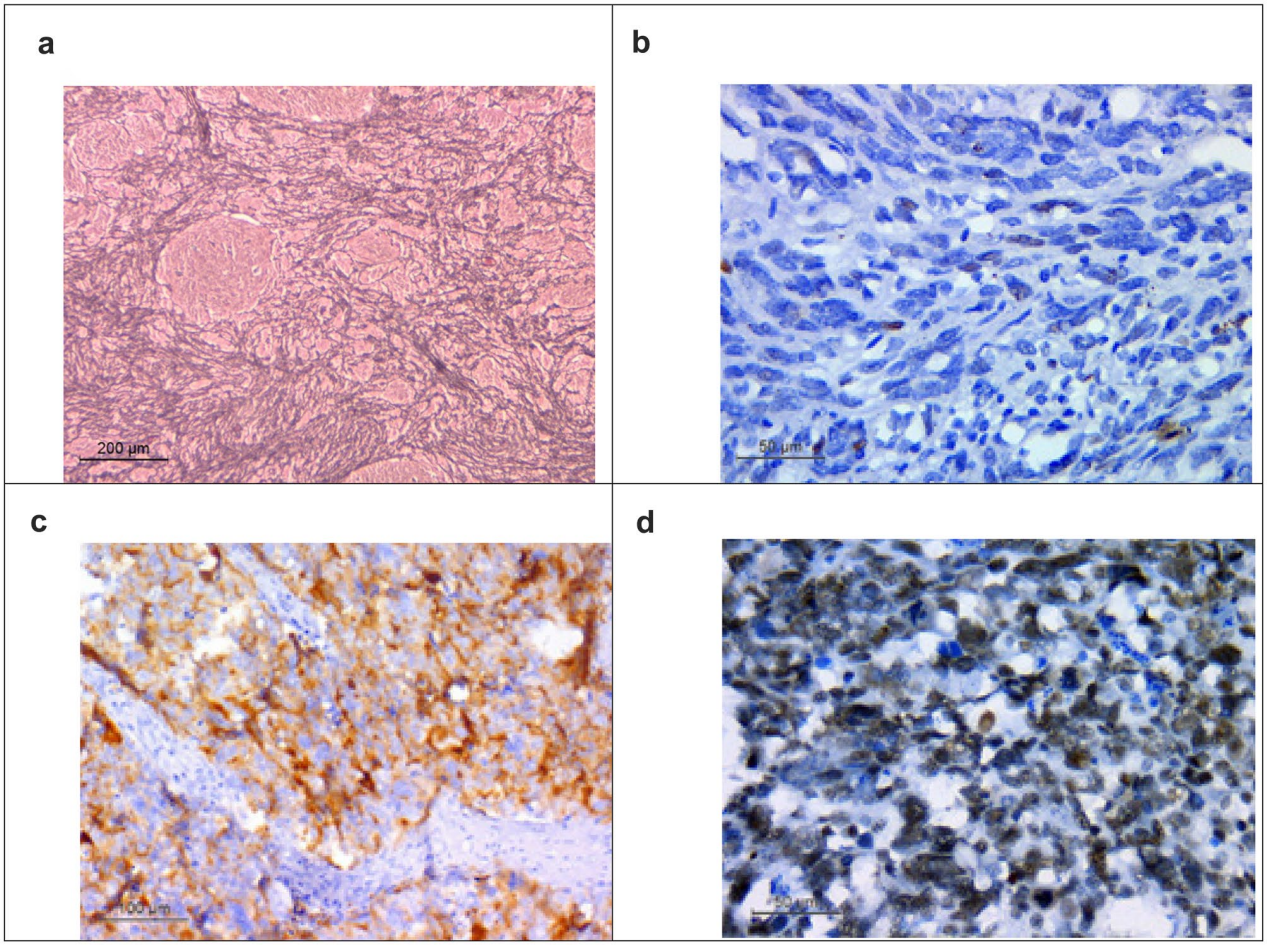
SHH-activated TP53-wildtype MB **a** the nodules are reticulin-free zones surrounded by a dense intercellular reticulin fiber network. (Reticulin stain X100) **b** p53 positive nuclear staining in scattered cells (wild type pattern) (anti p53 X400). Non-WNT/non-SHH MB, LC/A histology **c** synaptophysin positive cytoplasmic staining (anti synaptophysin X200) **d** retained INI1 nuclear staining (anti INI1 X400)

35 cases (70%) showed classic histology, while 13 cases (26%) were DNMB, and only 2 cases (4%) showed LC/A histology. One case (2%) belonged to the WNT group, the SHH-activated and TP53-wild-type group represented 13 cases (26%), and 36 cases (72%) belonged to the non-WNT/non-SHH group (Figs. 1 and 2). Clinicopathological characteristics of patients based on molecular classification are listed in Table 1. Statistical analysis showed that histology and preoperative size of the tumor (cm^2^) showed significant relation to molecular groups (*p* =  < 0.001 and 0.03, respectively), where all SHH-activated and TP53-wild-type groups were associated with DNMB histology (100%), and the non-WNT/non-SHH group was associated with classic histology in 94.4% of cases. The preoperative size of tumor in the SHH-activated and TP53-wild-type group (*Mdn* = 22.5) was significantly larger than in the non-WNT/non-SHH group (*Mdn* = 16).

**Table 1:** Relation between clinicopathological criteria and molecular groups

	Non-WNT/non SHH	SHH-activated and TP53-wild-type	Test (*p* value)
Age			
≤ 3y	4 (11.10)	1 (7.7)	*FEP* = 1
> 3 y	32 (88.90)	12 (92.3)	
Preoperative size (cm^2^)			
* M* ± *SD*	16	22.5	0.03*
(Min–max)	(9–45)	(16–33.6)	
Leptomeningeal dissemination			
Yes	11 (30.6)	4 (30.8)	*FEP* = 1
No	25 (69.40)	9 (69.2)	
Risk stratification			
High risk	22 (61)	5 (38.5)	0.159
Standard risk	14 (39)	8 (61.5)	
Histologically defined MB			
Classic MB	34 (94.4)	0 (0)	< 0.001*
DNMB	0 (0)	13 (100)	
LC/A MB	2 (5.6)	0 (0)	
Postoperative residual mass			
No residual	22 (62)	9 (69.2)	0.657
< 1.5 cm^2^	4 (12)	2 (15.4)	
> 1.5 cm^2^	10 (23)	2 (15.4)	

All tests were by Pearson’s chi-square, while preoperative size was by Mann–Whitney *U* test

*FEP* Fisher’s exact significance, *M* mean, *SD* standard deviation, *MB* medulloblastoma, *DNMB* desmoplastic nodular medulloblastoma, *LC/A* large cell anaplastic

*Statistically significant

One case belonged to WNT molecular group; the patient was 15 years old, and the cross-sectional area of the tumor was 13.5cm^2^, high risk with postoperative residual > 1.5 cm^2^, and showed classic histology. For statistical reasons, this case was not included in statistical analysis Fig. 1.

### MicroRNA-125a expression in MB patients 

2^−∆CT^ ranged between 0.000019 and 324 (*Mdn* = 8.3).

### Relation between microRNA-125a expression and clinicopathological parameters

**Fig. 3:**
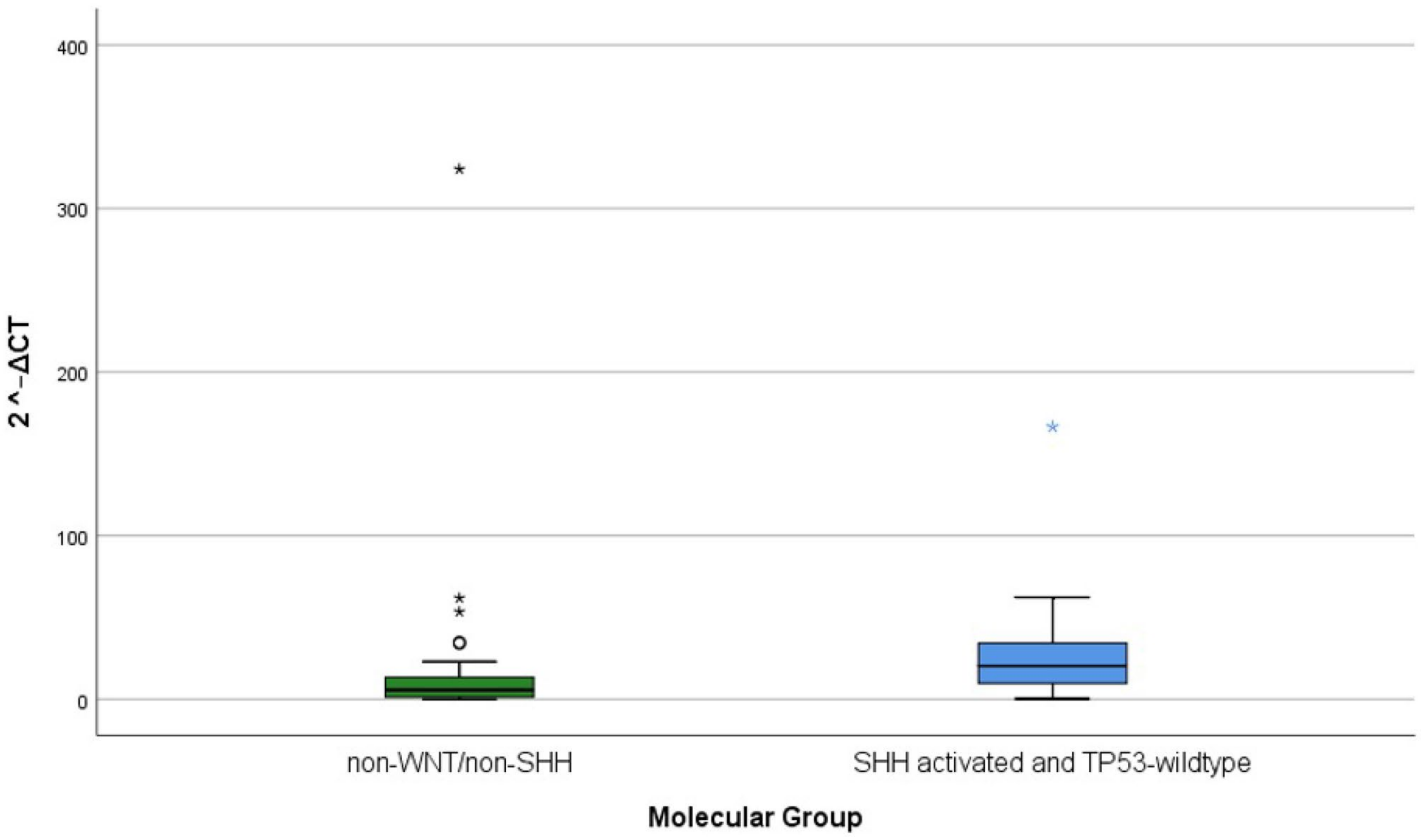
Box plot showing microRNA-125a expression according to MB molecular groups: non-WNT/non-SHH (n=36) and SHH-activated & TP53-wildtype (n= 13); center line= median. (p= .026 according to Mann Whitney U test)

MicroRNA-125a levels showed significant relation with histological patterns and molecular groups. The levels of microRNA-125a were significantly lowest in cases with LC/A histology (*Mdn* = 2.2) followed by classic histology (*Mdn* = 6) followed by DNMB (*Mdn* = 20) (*p* = 0.049). The levels of microRNA-125a in the non-WNT/non-SHH group (*Mdn* = 5.6) were significantly lower than the levels of microRNA-125a in the SHH-activated and TP53-wild-type group (*Mdn* = 20.3) (*p* = 0.026) (Table 2, Fig. 3).

**Table 2:** Relation between microRNA-125a (2^−ΔCT^) expression and risk stratification, leptomeningeal dissemination, histologically defined MB, and molecular groups

	2^−ΔCT^				Test (*p* value)
	*Mdn*	(Min–max)			
Risk stratification:					
High risk	10	(0.00001–324)			*U* = 0.423
Standard risk	7.5	(0.2–56)			
Leptomeningeal dissemination					
Yes	5	(0.000019–166)			*U* = 0.34
No	9.6	(0.2–324)			
Histologically defined MB					
Classic MB	6			(0.00001–324)	*H* = 0.049*
DNMB	20			(0.26–166.3)	
LC/A MB	2.2			(0.3–4)	
Molecular groups					
Non-WNT/non SHH MB	5.6		(0.000019–324)		*U* = 0.026*
SHH-activated and TP53-wild-type	20.3		(0.26–166.5)		

All tests were by Mann–Whitney *U* test (*U*), while histologically defined MB was by Kruskal–Wallis test (*H*)

*Mdn* median, *MB* medulloblastoma, *DNMB* desmoplastic nodular medulloblastoma, *LC/A* large cell anaplastic

*Statistically significant

There was no statistically significant correlation between levels of microRNA-125a and either preoperative size of the tumor (*rs* = 0.314 and *p* = 0.136) or age of the patients (*rs* =  − 0.265 and *p* = 0.063).

### Survival analysis

Median follow-up time was 37 months. The number of deaths in patients included in the study was 23 (46%). The 5-year OS was 60% with a mean of 77 months and a median of 69 months (95% CI, 61.873–92.418). The number of events was 28 (56%). The 5-year EFS was 42% with a mean of 64.5 months and a median of 39 months (95% CI, 48.821–80.318).

### Effect of microRNA-125a expression on clinical outcome of MB patients

**Fig. 4:**
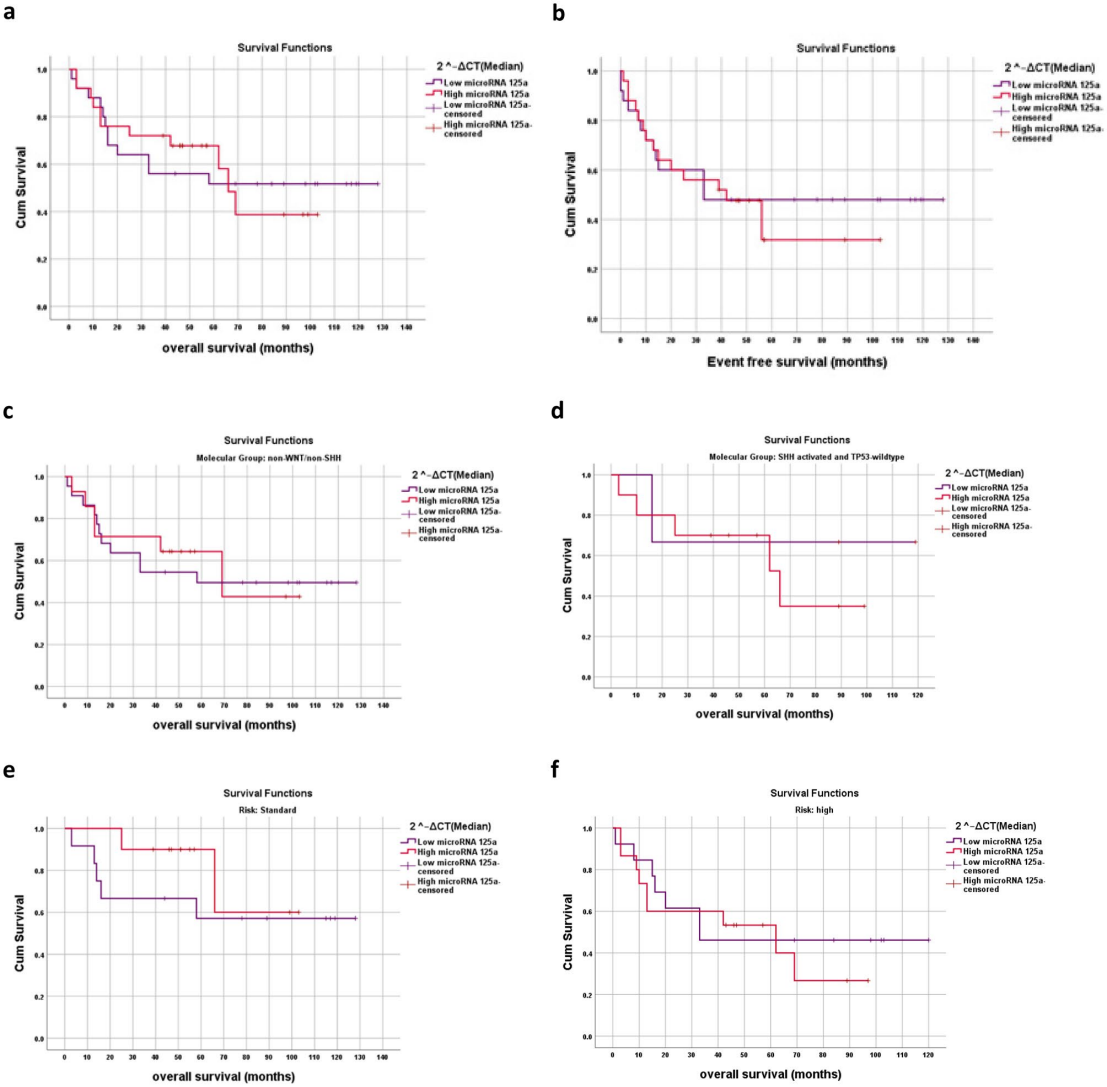
Kaplan–Meier survival curves for microRNA-125a expression in pediatric MBs. Patients were sub-divided into high and low microRNA-125a expression groups based on the median expression values. **a** and **b **OS and EFS for microRNA-125a in the 50 MB patients under the study, **c** OS for microRNA-125a expression in non-WNT/non-SHH molecular group, **d** OS for microRNA-125a expression in SHH-activated & TP53-wildtype molecular group, **e** OS for microRNA-125a expression in standard-risk group, **f** OS for microRNA-125a expression in high-risk group

MicroRNA-125a expression was divided into low- and high-expression levels in relation to median. The 5-year OS tends to be lower in the cases with low microRNA-125a than the cases with high microRNA-125a (52% versus 70%). The cases with low microRNA-125a levels showed a tendency for lower EFS (*Mdn* = 33 months) than the cases with high microRNA-125a levels (*Mdn* = 42 months). Neither of the differences in OS and EFS were significant (*X*^2^ = 0.000 and 0.189, *p* = 1 and 0.664 respectively) (Table 3, Fig. 4a, b).

**Table 3:** Relation of overall survival (OS) and event free survival (EFS) to different clinicopathological parameters

	OS				EFS				
	Mean	Median	5-year %	*p* value	Mean		Median	5-year %	*p* value
Age									
≤ 3 years	46	14	40%	0.314	12.4		6		0.012*
> 3 years	79.361		60%		68.729		56	45%	
Postoperative residual mass									
No residual mass	80		65%	0.109	69.172			50%	0.245
< 1.5 cm^2^	92.167		70%		69.5		25	50%	
> 1.5 cm^2^	44.692	42	47%		37.051		20	20%	
Leptomeningeal dissemination									
Yes	53.6	33	50%	0.266	37		15	30%	0.075
No	82.355		65%		72.992		56	50%	
Histologically defined MB									
Classic MB	80.38		60%	0.242	66.371		42	45%	0.119
DNMB	73.846	66	70%		64.754		56	45%	
LC/A MB	18	16			8.5		7		
Risk stratification									
High risk	62.371	42	50%	0.083	48.393		15	32%	0.056
Standard risk	91.936		70%		82.182			57%	
Molecular groups									
Non-WNT/non-SHH	75.847	69	55%	0.862		61.611	33	40%	0.678
SHH-activated and TP53-wild-type	73.846	66	70%			64.754	56	42%	
MicroRNA 125a (median)									
Low levels	75.545		52%	0.998		68.24	33	50%	0.664
High levels	63.774	66	70%			49.489	42	30%	

*MB* medulloblastoma, *DNMB* desmoplastic nodular medulloblastoma, *LC/A* large cell/anaplastic

*Statistically significant

### Relations of OS and EFS to different clinicopathological parameters (Table 3)

Kaplan–Meier curves revealed that EFS in patients ≤ 3 years of age (*Mdn* = 6 months) was significantly lower than patients > 3 years of age (*Mdn* = 56 months), (*X*^2^ = 6.3, *p* = 0.012). Univariate Cox regression showed that each unit increase in size of the tumor seems to increase the hazard of the event both for OS and EFS; this was significant in EFS (EFS: *HR* = 1.078, *p* = 0.031), but it was not significant in OS (OS: *HR* = 1.062, *p* = 0.165).

### Multivariate Cox model

It was then performed to identify the relationship between prognosis and the following covariates: microRNA-125a gene expression, age of the patients, and preoperative tumor size. Although the increased microRNA-125a gene expression and older age of the patients seemed to be protective against the event in both OS and EFS, this relation was not statistically significant (OS: *HR* = 0.870 and 0.265, *p* = 0.607 and 0.266 respectively; EFS: *HR* = 0.943 and 0.492, *p* = 0.792 and 0.524 respectively). Each unit increase in size of the tumor seemed to increase the hazard of the event both for OS and EFS; this was statistically significant in EFS (EFS: *HR* = 1.085, *p* = 0.040) (Table 4).

**Table 4:** Multivariate Cox proportional hazards analysis

	OS		EFS	
	HR	*p* value	HR	*p* value
MicroRNA-125a gene expression (2^−ΔCT^)	0.870	0.607	0.943	0.792
Age at diagnosis	0.265	0.266	0.492	0.524
Preoperative tumor size (cm^2^)	1.078	0.136	1.085	0.040*

*OS* overall survival, *EFS* event free survival, *HR* hazard ratio

*Statistically significant

### Evaluation of microRNA-125a gene expression within molecular and risk groups

Low levels of microRNA-125a expression showed a probability of poor outcome within the non-WNT/non-SHH group (50% 5-year OS for cases with low microRNA-125a versus 65% 5-year OS for cases with high microRNA-125a). However, the difference was not statistically significant (*X*^2^ = 0.041, *p* = 0.840). Conversely, within the SHH-activated and TP53-wild-type group, the 5-year OS for cases with low microRNA-125a (67%) was close to those with high microRNA-125a (70%). This result was not statistically significant (*X*^2^ = 0.398, *p* = 0.528) (Fig. 4c, d).

Low levels of microRNA-125a expression were possibly associated with poor outcome within both standard-risk group (58% 5-year OS for cases with low microRNA-125a versus 90% 5-year OS for cases with high microRNA-125a) and high-risk group (45% 5-year OS for cases with low microRNA-125a versus 55% 5-year OS for cases with high microRNA-125a). However, the differences were not statistically significant (*X*^2^ = 0.784 and 0.236, *p* = 0.376 and 0.627 respectively) (Fig. 4e, f).

## Discussion

MB is the most common malignant pediatric brain tumor [23]. Advances in molecular profiling identified four molecular groups that guided clinical decision [8]. However, in a recent study, seven molecular subgroups were identified. While WNT remained the same, SHH, group 3, and group 4 were split into further groups, hence reflecting heterogeneity at the molecular level which brings challenge to understanding the biology of MB with impact on treatment options [24]. The current treatment regimens show considerable morbidity. Thus, numerous treatment strategies are designed and tailored in light of our molecular understanding of MBs [25].

In the present work, 5-year OS was 60% which is consistent with those reported in the literature (50–70%) [26]. Age showed a prognostic role where children ≤ 3 years old conferred poor survival, in accordance with findings observed by Zeltzer et al. [27].

It was also found in a uni- and multivariate analysis that preoperative tumor size represents an independent prognostic factor, where the larger the tumor, the poorer the prognosis with significant relation to EFS rates. Our findings are similar to those reported by Evans et al. [28].

MicroRNAs play important roles in regulating gene expression acting as either oncogenes or tumor suppressors under certain conditions [29].

MicroRNA-125a expression is downregulated in several tumors and was found to be a prognostic marker and a potential for therapeutic target in some of these tumors [12, 13].

In a single study, conducted before the release of the 2016 WHO classification, Ferretti et al. found that most microRNAs displayed overall downregulated expression in MB [11].

The current study is the first study to report the expression profile and clinical significance of microRNA-125a in molecular groups of MB and to evaluate its prognostic significance. Fifty MB tumors were collected retrospectively and classified into three molecular groups. The relation between microRNA-125a and different clinicopathological parameters showed statistically significant relation to histology and molecular groups, while there was no significant relation to age or tumor size. MicroRNA-125a was significantly lower in MB cases with LC/A histology followed by classic histology and then DNMB. It was also significantly lower in non-WNT/non-SHH molecular group than SHH-activated and TP53-wild-type. The one WNT MB case could not be included in statistical analysis.

In the present work, it was found that lower levels of microRNA-125a were associated with parameters described in the literature to have worse prognosis namely LC/A histology and non-WNT/non-SHH molecular group suggesting a pathogenetic role for microRNA-125a in these tumors. Huang et al. stated that LC/A histology was one of the most significant factors associated with worse survival rates [30].

In the current study, SHH-activated and TP53-wild-type MB showed 70% 5-year OS, while non-WNT/non-SHH MB showed 55% 5-year OS, consistent with the findings in literature where SHH-activated and TP53-wild-type MB proved to have a favorable outcome [31], while non‐WNT/non‐SHH tumors have the worst prognosis overall [32].

The lower levels of microRNA-125a in categories with worse prognosis could be explained by the findings in literature suggesting that it has tumor suppressor function through inhibiting proliferation, invasiveness, and metastasis [33]. It was found to be downregulated in different tumors, where its upregulation by targeted therapy induced apoptosis. In neuroblastoma, it was found to interfere with the expression of proproliferative truncated isoform of the neurotrophin receptor tropomyosin–related kinase C (t-trkC), where its underexpression was associated with poor prognosis [34]. In glioblastoma, microRNA-125a is downregulated where it targets podoplanin and inhibits cell migration and invasion [35]. According to Ferretti et al., specific microRNA signatures distinguished tumors from normal cerebellar tissues. Most microRNAs displayed overall downregulated expression in MB, which rescued expression promotes MB cell growth arrest and apoptosis while targeting the proproliferative t-TrkC isoform, suggesting a tumor growth–inhibitory function [11]. Therefore, microRNA-125a could represent a potential target for targeted therapy, a feature observed in clinical trials in different tumors [13, 36, 37].

Non-WNT/non-SHH molecular group which includes group 3 and group 4 remains the genetically most heterogeneous and least understood fraction of MB cases [38]. In contrast to the WNT and SHH groups, no single somatically mutated gene is present in more than 5–10% of either group 3 or group 4, constituting a significant challenge in the development of innovative treatment strategies for these groups [8]. The significantly lower levels of microRNA-125a in the non-WNT/non-SHH group provide a chance for targeted therapy aiming at increasing the levels of microRNA-125a in these patients.

In the current study, the cases with lower levels of microRNA-125a had lower 5-year OS than the cases with higher levels, and possibly, these cases will have poor outcome, but the difference was not statistically significant. This could be explained by the fact that it acts as tumor suppressor gene and is consistent with the reported poor survival rates of reduced levels of microRNA-125a in different tumors [39, 40].

In the present study, whether microRNA-125a expression could be helpful in segregating patients with poor outcome within both risk groups was assessed. Although standard-risk patients with lower expression levels have lower 5-year OS 58% versus 90% for patients with higher expression levels, the difference was not significant. The effect of stratifying patients based on microRNA-125a expression within each molecular group was also examined and showed that patients with lower levels had lower 5-year OS in non-WNT/non-SHH molecular group. This was not the case in SHH molecular group. These differences were not significant. The current study suggests that microRNA-125a might be helpful in predicting patients’ outcome; although the differences in survival rates in the present work were not statistically significant which can be explained by the small sample size, the findings are promising in this context and need to be validated in larger groups.

## Conclusion

MicroRNA-125a expression was significantly lower in categories of pediatric MB patients with worse prognosis namely LC/A histology and non-WNT/non-SHH molecular group suggesting a pathogenetic role. MicroRNA-125a expression could represent a promising prognostic factor and a potential therapeutic target in non-WNT/non-SHH molecular group which represents the most common and the most heterogeneous group of pediatric MBs coupled with the highest rates of disseminated disease. Preoperative tumor size represents an independent prognostic factor.

## Data Availability

The datasets generated during and/or analysed during the current study are available from the corresponding author on reasonable request.
